# A Quantum Vaccinomics Approach for the Design and Production of MSP4 Chimeric Antigen for the Control of *Anaplasma phagocytophilum* Infections

**DOI:** 10.3390/vaccines10121995

**Published:** 2022-11-24

**Authors:** José de la Fuente, Alberto Moraga-Fernández, Pilar Alberdi, Sandra Díaz-Sánchez, Olga García-Álvarez, Rubén Fernández-Melgar, Marinela Contreras

**Affiliations:** 1 SaBio, Instituto de Investigación en Recursos Cinegéticos (IREC), Consejo Superior de Investigaciones Científicas (CSIC), Universidad de Castilla-La Mancha (UCLM)-Junta de Comunidades de Castilla-La Mancha (JCCM), Ronda de Toledo s/n, 13005 Ciudad Real, Spain; 2 Department of Veterinary Pathobiology, Center for Veterinary Health Sciences, Oklahoma State University, Stillwater, OK 74078, USA; 3 Ciudad Real Medical School, Regional Center for Biomedical Research, University of Castilla La Mancha, 13005 Ciudad Real, Spain; 4 Departamento de Bioquímica, Microbiología, Biología Celular y Genética, Área de Microbiología, Entrada Campus Anchieta, 4, Universidad de La Laguna, 38200 La Laguna, Tenerife, Canary Islands, Spain; 5 ETSIA Ciudad Real (UCLM), 13071 Ciudad Real, Spain

**Keywords:** tick, vaccine, sheep, *Anaplasma phagocytophilum*, rabbit, epitope, microarray, quantum vaccinomics

## Abstract

*Anaplasma phagocytophilum* Major surface protein 4 (MSP4) plays a role during infection and multiplication in host neutrophils and tick vector cells. Recently, vaccination trials with the *A. phagocytophilum* antigen MSP4 in sheep showed only partial protection against pathogen infection. However, in rabbits immunized with MSP4, this recombinant antigen was protective. Differences between rabbit and sheep antibody responses are probably associated with the recognition of non-protective epitopes by IgG of immunized lambs. To address this question, we applied quantum vaccinomics to identify and characterize MSP4 protective epitopes by a microarray epitope mapping using sera from vaccinated rabbits and sheep. The identified candidate protective epitopes or immunological quantum were used for the design and production of a chimeric protective antigen. Inhibition assays of *A. phagocytophilum* infection in human HL60 and *Ixodes scapularis* tick ISE6 cells evidenced protection by IgG from sheep and rabbits immunized with the chimeric antigen. These results supported that the design of new chimeric candidate protective antigens using quantum vaccinomics to improve the protective capacity of antigens in multiple hosts.

## 1. Introduction

*Anaplasma phagocytophilum* (Rickettsiales: Anaplasmataceae) is a tick-borne intracellular bacterial pathogen emerging in many regions of the world where it causes human granulocytic anaplasmosis (HGA), tick-borne fever (TBF) and canine anaplasmosis [1]. *A. phagocytophilum* infection has been documented in a wide host range including cattle, goat, sheep, horse, dog, human, roe deer (*Capreolus capreolus*), red deer (*Cervus elaphus*), white-tailed deer (*Odocoileus virginianus*) and several rodents (*Apodemus sylvaticus*, *Microtus agrestis*, *Clethrinomyces glareolus*, *Peromyscus* spp., *Zapus hudsonius*, *Clethrionomys gapperi*, *Microtus* spp., *Tamias* spp., *Spermophilus lateralis*, *Sigmodon hispidus*, *Sciurus* spp., *Neatoma* spp.) [1,2].

The clinical signs associated with *A. phagocytophilum* vary from mild to severe in the different species. Among the clinical manifestations, lethargy and fever are the most common, and reluctance to move, lameness, polydipsia, anemia, pale mucous membranes, diarrhea, vomiting, petechia, hemorrhage, splenomegaly and enlarged lymph nodes may also occur but depending on the strain and the susceptibility and immune status of the host [3,4,5,6]. In human patients, the clinical range of HGA extends from asymptomatic infection to fatal disease, and a direct correlation between patient age and illness severity is reported [7]. In humans, doxycycline hyclate has been reported as the agent of choice for treatment with a clinical improvement in 24–48 h and recovery [7]. In other species, prophylactic uses of tetracycline together with acaricide applications for tick control are the main measures to control *A. phagocytophilum* infection, especially in endemic areas [8]. However, these control measures may cause an impact on the environment and human health, and the selection of resistant pathogens and ticks [5,6,7,8].

Vaccination is a promising alternative for the control of tick-borne diseases because it is environmentally friendly by reducing acaricide and antibiotic use and constitutes the safest and effective intervention. Vaccines producing a long-lasting immunity could prevent or reduce tick infestations and pathogen transmission [9,10,11]. Currently, vaccines are not available for the prevention and control of *A. phagocytophilum*. In the control of tick-borne pathogens, the tick protective antigen Subolesin (SUB) appears to be a candidate antigen that may contribute to the control of multiple tick species and the reduction of tick-borne pathogens [10,11].

One of the main limitations for the development of effective vaccines for the prevention and control of *A. phagocytophilum* infection and transmission is the identification of effective protective antigens. Studying the molecular interactions between ticks and transmitted pathogens would facilitate the identification of candidate tick antigens to reduce pathogen infection and transmission while also affecting tick infestations. Recent results have shown how the application of a vaccinomics approach to *Ixodes scapularis-A. phagocytophilum* interactions allowed the identification and characterization of candidate tick protective antigens for the control of vector infestations and *A. phagocytophilum* infection [12,13]. Studying the proteins involved in the processes by which *A. phagocytophilum* establishes infection, it has been found that the major surface protein 4 (MSP4) localized on the bacterial membrane plays a role during pathogen infection in ticks [14], and would be a possible vaccine target, characterizing its potential protective capacity in immunized animals. 

Previous results provided the first evidence for the role of *A. phagocytophilum* MSP4 and heat-shock protein 70 (HSP70) proteins during infection and multiplication in host neutrophils [15]. In particular, MSP4 is involved in the interaction with host cells and may be used to develop double-effect vaccines targeting infection in both vertebrate hosts and tick vectors [15]. Previous vaccination trials in sheep with the *A. phagocytophilum* MSP4 showed only partial protection in lambs and differences between rabbit and sheep antibody responses are probably associated with the recognition of non-protective epitopes by IgG of immunized lambs [16]. 

Beyond that, the application of quantum vaccinomics allows the characterization of vector–host–pathogen molecular interactions using omics technologies combined with multiomics data integration and analysis [17]. Results could be used for the identification and characterization of the protective epitopes or immunological quantum for the design and production of chimeric protective antigens that otherwise do not develop a strong protective immune response when using the entire antigen in the vaccine formulation.

## 2. Materials and Methods

### 2.1. Antibody IgG Binding to MSP4 Epitopes Microarray and Data Analysis

Peptide MSP4 microarray elongated with neutral GSGSGSG linkers at the C- and N-terminus and translated into 282 different overlapping 15 aa peptides was printed in duplicate (564 peptide spots each array copy). Sera from sheep and rabbits immunized with MSP4 from a previous vaccination trial were pooled (n = 3) and used to identify candidate protective regions or epitopes in *A. phagocytophilum* MSP4 (GenBank ID: AFD54597) to increase vaccine efficacy by applying quantum vaccinomics. A high-resolution epitope mapping of *A. phagocytophilum* MSP4 protein was performed (PEPperCHIP^®^ Immunoassay, PEPperPRINT, Germany). The peptide microarray was assembled in an incubation tray and blocked with 1% (*w/v*) bovine serum albumin (BSA) in 1× PBS 7.4 with 0.005% (*v*/*v*) Tween-20 (PBST) for 30 min at room temperature. After it was washed with PBST three times, the array was incubated with pooled sera overnight at 4 °C. The next day, it was washed again, and the array was incubated with a goat anti-rabbit IgG (H + L)-Alexa Fluor 532 nm (Thermo Fisher Scientific, Waltham, MA, USA) antibody, and donkey anti-sheep IgG (H + L)-Cy3 antibody (Sigma-Aldrich , St. Louis, MO, USA), for 45 min at room temperature (RT). The array was washed, dissembled from the tray, and dried with centrifugation for 2 min at 2000 rpm. The resulting array was scanned with a GenePix personal 4100a microarray scanner (Molecular Devices, San José, CA, USA). The median fluorescent signal intensity of each spot was extracted using MAPIX software (Molecular Devices). Candidate protective epitopes were identified by IgGs from sera of rabbits and lambs vaccinated with the recombinant protein MSP4 in a previous study [16] using this method [17].

For data analysis, the intensity of the raw fluorescence signal that corresponded to the median signal intensity subtracted by the median background intensity of each spot was considered and then averaged across duplicate spots. Epitopes significantly recognized by IgG were defined as amino acids shared by overlapping peptides and normalized with a Z-score [18,19]. Additionally, a heatmap (http://www.heatmapper.ca/expression/ (accessed on 4 March 2022) of IgG antibody binding to the peptides was visualized where peptides that showed Z-scores > 2 were considered significant reactive peptides and were used to express a MSP4 chimeric antigen for the control of anaplasmosis containing the epitopes or regions that showed protection against the pathogen in rabbits (peptide+) and another peptide containing the non-protective region found in lambs immunized with MSP4 (peptide−) was also expressed.

### 2.2. Production and Characterization of the Recombinant Chimeric Antigen and Vaccine Formulation

The coding sequences for new candidate protective chimeric antigens were amplified from synthetic genes optimized for codon usage in *Escherichia coli* (Genscript Corporation, Piscataway, NJ, USA) with sequence-specific primers using the *A. marginale* MSP1a chimera expression system previously reported [20]. The inserted coding region is fused to MSP1a and is under the control of the inducible tac promoter [21]. Recombinant BL21 *E. coli* were propagated in 1 l flasks containing 200 mL Luria–Bertani (LB) broth supplemented with 10 g tryptonel−1, 5 g yeast extract l^−1,^ 10 g NaCl l^−1^, 50 µg/mL ampicillin, and 0.5% glucose (VWR, Radnor, PA, USA) for 2 h at 37 °C and 200 rpm and then for 5 h after addition of 0.5 mM final concentration of isopropyl-ß-D-thiogalactopyranoside (IPTG) for induction of recombinant protein [21]. Cell growth was monitored by measuring OD at 600 nm. The cells were harvested by centrifugation at 3900× *g* for 15 min at 4 °C, and then 1 g of cell pellet was resuspended in 5 mL of disruption buffer (100 mM Tris–HCl, pH 7.5, 150 mM NaCl, 1 mM PMSF, 5 mM MgCl_2_·6H_2_O and 0.1% (*v*/*v*) Triton X-100) and disrupted using a cell sonicator (Model MS73; Bandelin Sonopuls, Berlin, Germany). After disruption, the insoluble fraction containing the membrane-bound MSP1a chimera antigens was collected by centrifugation at 12,000× *g* for 15 min at 4 °C and stored at −20 °C until characterization and vaccine formulations. Protein concentration was determined using bicinchoninic acid (BCA) (Thermo Fisher Scientific, Waltham, MA, USA) following the manufacturer’s recommendations. For vaccine formulation, recombinant proteins were adjuvated in Montanide ISA 50 V2 (Seppic, Paris, France), to a final protein concentration of 100 µg/mL [14].

### 2.3. Western Blot Analysis of Recombinant Chimeric Protein Expression 

Ten micrograms of each recombinant protein and insoluble *E. coli* fraction were loaded onto a 12% SDS-polyacrylamide pre-cast gel (Genscript Corporation, Piscataway, NJ, USA) and transferred to a nitrocellulose membrane. The membrane was blocked with 5% bovine serum albumin (BSA) (Sigma-Aldrich, St. Louis, MI, USA) for 1.5 h at RT and washed three times with TBS-T (50 mM Tris-Cl, pH 7.5, 150 mM NaCl, 0.5% Tween 20). Purified IgG from rabbits immunized with MSP1a at 1:500 dilution and serum samples from preimmunized and immunized sheep and rabbits were used and 1:250 dilutions in TBS respectively to each incubation membrane. The membranes were incubated overnight at 4 °C and then washed four times with TBS-T. After that, the membranes were incubated with an anti-rabbit IgG-horseradish peroxidase (HRP) conjugate (Sigma-Aldrich) or an anti-sheep IgG-peroxidase (HRP) conjugate (Sigma-Aldrich) diluted 1:1000 in blocking solution (TBS with 3% BSA). The membranes were washed four times with TBS-T and finally developed with TMB (3,3′, 5,5′- tetramethylbenzidine) stabilized substrate for HRP (Promega, Madrid, Spain) according to the manufacturer’s recommendations. 

### 2.4. Immunization in Sheep and Rabbits

Six 1-year old sheep were selected and two groups of 3 sheep each were formed, from the experimental sheep flock maintained at NEIKER (Alava, Spain), with similar live weights being formed. One group was immunized with the chimeric antigen containing the epitopes recognized by IgGs from MSP4-immunized rabbits (peptide+), and the other group of sheep was immunized with the chimeric antigen containing the epitope recognized by IgGs from the previous MSP4-immunized lambs (peptide−) [16]. Sheep from each group were injected subcutaneously with 100 µg in 1 mL dose of the antigens in the loose skin of the axilla (armpit) using a sterile syringe with a removable needle 20 G × 1′′ (9.0 × 25 mm) under aseptic conditions. Sheep were immunized three times on days 0, 20, and 55 and blood samples were collected from the jugular vein of each sheep before each immunization and at the end of the experiment for serum preparation [16]. An additional immunization was conducted in rabbits to confirm the effect of the protective chimera (peptide+) in this host. Two groups of six rabbits each were immunized by injecting subcutaneously using a 1-mL tuberculin syringe and a 22-G needle three times at weeks 0, 2, and 4 with 0.5 mL doses (50 μg) but using recombinant peptide + and adjuvant/saline alone as a control. Blood was collected at times 0 and 2 weeks after the last immunization and used for serum preparation.

### 2.5. Antibody Inhibition Assay with IgG from Immunized Sheep

Samples were prepared from *Ixodes scapularis* embryo-derived tick cells (ISE6) grown in L15B300 medium and the human promyelocytic HL-60 cells maintained in RPMI1640 medium supplemented with 10% heat-inactivated fetal calf serum, 2 mM L-glutamine and 25 mM Hepes buffer at 36.5 °C in a humidified atmosphere containing 5% CO_2_ (3 replicates each). The inhibitory effect of IgG antibodies from MSP4 immunized lambs and rabbits (IgGs are from a previous study [16]) and from sheep immunized with the chimeric antigens (peptide+ and peptide−) 15 days after the third immunization on *A. phagocytophilum* human NY18 was evaluated using purified sera IgGs using the NAb Protein G spin kit (Thermo Fisher Scientific, Waltham, MA, USA) following manufacturer’s recommendations. Sheep and rabbit IgG antibodies from pre-immune sera were used as a control [20,22]. Each cell line, ISE6 and HL60, was pooled separately and used to seed 24-well plates. In addition, 1 × 10^6^ cells were seeded in each well 48 h prior to inoculation with *A. phagocytophilum* human NY18 isolate. Rabbit or sheep purified IgGs (2 mg/mL) were mixed with the inoculum (1:1) for 120 min before being placed on the cell monolayers. Each monolayer then received 100 μL of the inoculum plus IgG mix and the plates were incubated at 34 °C for 60 min. The inoculum was removed from the wells and cell monolayers were washed three times with PBS. Complete medium (1 mL) was added to each well, then the ISE6 plates were incubated at 34 °C and HL60 plates were incubated at 37 °C. After 7 days, cells from all wells were recovered and frozen at −80 °C until *A. phagocytophilum* detection by PCR after DNA extraction using TriReagent (Sigma-Aldrich) according to the manufacturer’s recommendations. *A. phagocytophilum* infection levels were determined by *msp4* real-time PCR with normalization against the level of tick 16S rRNA in ISE6 DNA samples and against human β-actin in HL-60 DNA samples (delta *Ct* method) as described previously [16,22] using oligonucleotide primers MSP4-L (5′-CCTTGGCTGCAGCACCACCTG-3′) and MSP4-R (5′-TGCTGTGGGTCGTGACGCG3′), with PCR conditions of 5 min at 95 °C and 35 cycles of 10 s at 95 °C, 30 s at 55 °C and 30 s at 60 °C. To analyze the infection levels, normalized *Ct* values were compared between peptide +/− IgG treated cells and controls (preimmune IgG treated cells) by the Student’s *t*-test with unequal variance (*p* = 0.05; n = 3 biological replicates).

### 2.6. Antibody Inhibition Assay with IgG from Immunized Rabbits

An additional inhibition assay on *A. phagocytophilum* human NY18 was performed using IgGs purified from rabbits immunized with peptide + to confirm that peptide+ antibodies produced in rabbits may block infection as well as MSP4. HL-60 cells were cultured, and the experiment was performed in the same conditions mentioned above for HL60 cells, and the same IgG antibody purification protocol was used from rabbit serum samples collected at times 0 and 15 days after the third immunization. *A. phagocytophilum* infection levels were determined by real-time PCR [16,22], and results were compared between treatments by the Student’s *t*-test with unequal variance (*p* = 0.05; N = 6 biological replicates).

### 2.7. Peptide Sequence Alignment and A. marginale MSP4 Chimeric Antigen Design

In order to study a possible cross-protection against other pathogens an alignment of the peptide + amino acid sequence obtained from microarray analysis was conducted using Blastp (https://blast.ncbi.nlm.nih.gov/Blast.cgi?PROGRAM=blastp&PAGE_TYPE=BlastSearch&LINK_LOC=blasthome (accessed on 17 January 2022). The MSP4 chimeric antigen search was performed using the compositional matrix adjustment method [23] for analysis as implemented in Blastp. A conserved domains analysis was performed using database CDSEARCH/cdd search with an E-value threshold of 0.01 using Blastp.

## 3. Results and Discussion

### 3.1. MSP4 Epitope Mapping and Antibody IgG Binding

**Figure 1:**
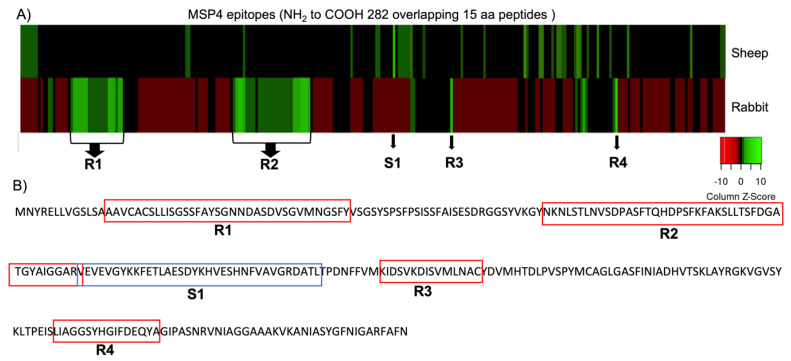
Reactive epitopes recognized by IgGs from rabbits and lambs immunized with *A. phagocytophilum* MSP4. (**A**) heatmap of the IgG reactive epitopes in rabbit and sheep. Peptides with significant Z-Score are indicated with R1 to R4 if they were recognized by rabbit IgG and S1 by sheep IgG; (**B**) reactive epitopes in MSP4 protein sequence. Red boxes indicate the epitopes recognized by rabbit IgG, and the blue box indicates an epitope recognized by sheep IgG. Reactive epitopes showed signal intensities with Z-score > 2.

The MSP4 epitope mapping was analyzed in a peptide microarray using the fluorescence intensity of immunoreactivity of IgG antibodies in sera from rabbits and lambs previously vaccinated with the recombinant protein MSP4 [16]. Then, the analysis was focused on reactive epitopes with Z-score > 2. A representative epitope heatmap is shown in Figure 1A where the Z-score is represented. Four reactive amino acid regions (R1, R2, R3, and R4), from *A. phagocytophilum* MSP4, showed overlapping peptides with Z-score > 2 when recognized by IgG antibodies from MSP4-immunized rabbits but only one significant peptide sequence (S1) was reactive to IgGs from an MSP4-immunized sheep. 

The analysis of distinctive reactive epitopes showed that IgG antibodies from rabbits immunized with MSP4 recognized immunogenic epitopes or overlapping amino acid regions that were different from peptides recognized by IgG from MSP4-immunized sheep (Table 1). 

Previous results showed that IgGs from rabbits immunized with MSP4 were potentially protective against *A. phagocytophilum* blocking the infection, but IgGs from immunized sheep did not provide protection against this pathogen [16]. Hence, the reactive epitopes identified in the MSP4 mapping by rabbit IgG recognition could be considered potentially protective or blocking. However, the reactive epitopes identified in MSP4 by IgGs from sheep did not provide protection against the pathogen. Previous studies have correlated reactive peptides identified by this approach to vaccine efficacy, suggesting that new chimeras could be designed [24]. Therefore, to improve the protection against *A. phagocytophilum* in sheep, a chimera containing the candidate protective epitopes identified in rabbits was designed. The chimera peptide + contained the four significant reactive regions identified with rabbit IgGs (AAVCACSLLISGSSFAYSGNNDASDVSGVMNGSFY-NKNLSTLNVSDPASFTQHDPSFKFAKSLLTSFDGATGYAIGGARV-KIDSVKDISVMLNAC-LIAGGSYHGIFDEQYA) (Table 1). Peptide—consisted of the single significant reactive region identified with sheep IgGs (VEVEVGYKKFETLAESDYKHVESHNFVAVGRDATL) (Table 1). 

An important point to be considered is that, usually, a potentially protective antigen is one that is antigenic but also immunogenic producing a specific immune response. This specific immune response or immunogenicity usually involves immunodominant domains, residues, or epitopes in a molecule, the rest of the molecule being ineffective at producing an immune response [25]. Herein, we mapped B-cell linear epitopes since the identification of these epitopes is essential in the development of efficient and target-specific vaccines. These protein regions are recognized by soluble or membrane antibodies and can contain either linear or discontinuous epitopes [26]. Linear epitopes that are not exposed in the native protein could also elicit strong immune responses producing antibodies [27,28], and the sequence of these epitopes can determine the three-dimensional conformation that confers the biological activity to the protein [29]. Therefore, the contribution of these antibodies to protection could be a relevant tool to study and control the immune responses; in addition, epitope recognition by B-cells and soluble antibodies constitute the core of the adaptive immune response [30,31]. Alternatively, it has been found that the application of this approach to the identification of protective B-cell epitopes would also allow the exclusion of any epitopes that could induce cross-reactive autoimmune antibodies [24,32].

Therefore, the results shown here provided the opportunity to predict specific epitopes or regions recognized by antibodies that, depending on the host, may be candidates potentially protective (rabbit) or non-protective (sheep) and could be used for the design and production of new and more effective chimeric antigen candidates [17,24] (Figure 1B) for the development of vaccines that induce enhanced immune responses with no adverse effects. 

### 3.2. Characterization of Candidate Chimeric Antigens

**Figure 2:**
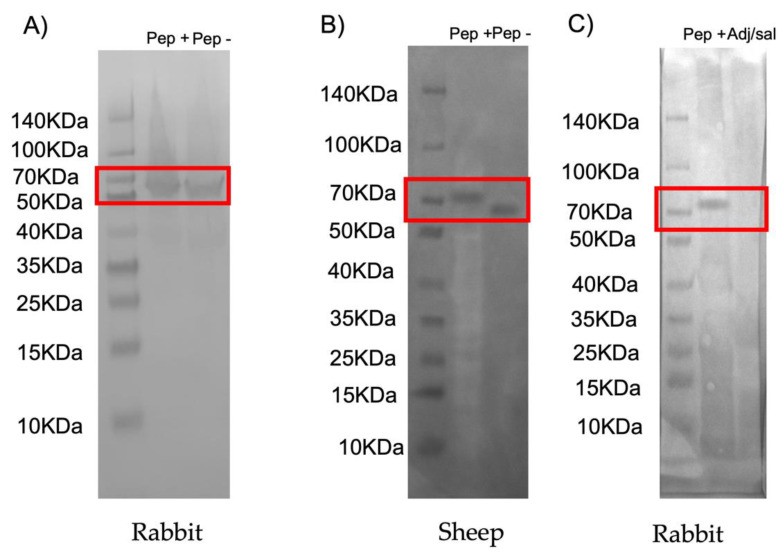
Production of the recombinant proteins and antibody detection of the chimeric proteins by IgG antibodies produced in sheep and rabbit previously immunized with MSP1a and the chimeric protein. Samples equivalent to 10 µg of total proteins were loaded on a 12% polyacrylamide gel and for Western blot analysis, proteins were transferred to a nitrocellulose membrane. (**A**) A Western blot was performed using IgG antibodies from rabbits immunized with recombinant MSP1a antigen in order to confirm the size of the chimeric MSP4 antigens. Peptide + (AAVCACSLLISGSSFAYSGNNDASDVSGVMNGSFY-NKNLSTLNVSDPASFTQHDPSFKFAKSLLTSFDGATGYAIGGARV-KIDSVKDISVMLNAC-LIAGGSYHGIFDEQYA) is the chimeric antigen that contained the epitopes or regions that showed protection against *A. phagocytophilum* in rabbits and showed a molecular weight of 73 kDa. Peptide- (VEVEVGYKKFETLAESDYKHVESHNFVAVGRDATL) contained the non-protective region found by IgGs from sheep immunized with MSP4 showing a molecular weight of 67 kDa. Red boxes denote the position of the recombinant antigens. Spectra (Thermo Fisher Scientific) was used as molecular weight markers for protein electrophoresis. (**B**) In the Western blot, proteins were incubated with sheep or rabbit antibodies against MSP4 chimeric proteins and developed with anti-sheep or anti-rabbit conjugate coupled to horseradish peroxidase. The position of the chimeric proteins is indicated by a red box. Spectra (Thermo Fisher Scientific) was used as molecular weight marker for protein electrophoresis. Peptide + and peptide- transferred to the nitrocellulose membrane were incubated with serum samples from sheep immunized with peptide+ (pep+) and peptide- (pep-). Both chimeric proteins were recognized at the expected molecular weight. (**C**) Peptide + transferred to the nitrocellulose membrane was incubated with serum samples from rabbit immunized with the peptide+ (pep+) and an adjuvant/saline formulation (adj/sal). Peptide+ was recognized at the expected molecular weight by pep + serum samples, and no signal was observed when the membrane was incubated with serum samples from rabbits immunized with PBS adjuvated (adj/sal).

The recombinant chimeric antigens were produced in *E. coli* using the MSP1a chimera expression system because the peptides fused to the *A. marginale* MSP1a N-terminal region are displayed on the *E. coli* surface and would be better recognized by the immune system [20]. These recombinant chimeras were used for the preparation of antigen-specific IgG antibodies in immunized sheep. To confirm the expression of the antigens, a Western blot was performed using IgG antibodies previously produced in rabbits immunized with the MSP1a protein, which is expressed bound to the peptides designed with the reactive epitopes obtained in the microarray. Their presence and detection confirmed the expression of the chimeric peptides at the predicted sizes of 73 kDa for the peptide+ corresponding to 11 kDa for the reactive peptide+ and 62 kDa for the MSP1a region and the predicted size of 67 kDa corresponding to the molecular weight of 5 kDa for the peptide- identified by sheep IgG and 62 kDa from MSP1a (Figure 2A and Figure S2). 

A second Western blot was also performed to characterize antigenically the recombinant peptides bound to MSP1a as a fusion protein using serum samples from sheep immunized with these chimeric antigens (peptide+ and peptide-) and rabbits immunized with peptide + and PBS adjuvated. These results indicated that surface-displayed peptide+ and peptide- epitopes on the fusion protein were recognized by the immune system of the sheep and rabbits and, therefore, the epitopes were translated correctly and maintained their antigenicity within the surface-exposed fusion protein (Figure 2B,C and Figure S2).

**Figure 3:**
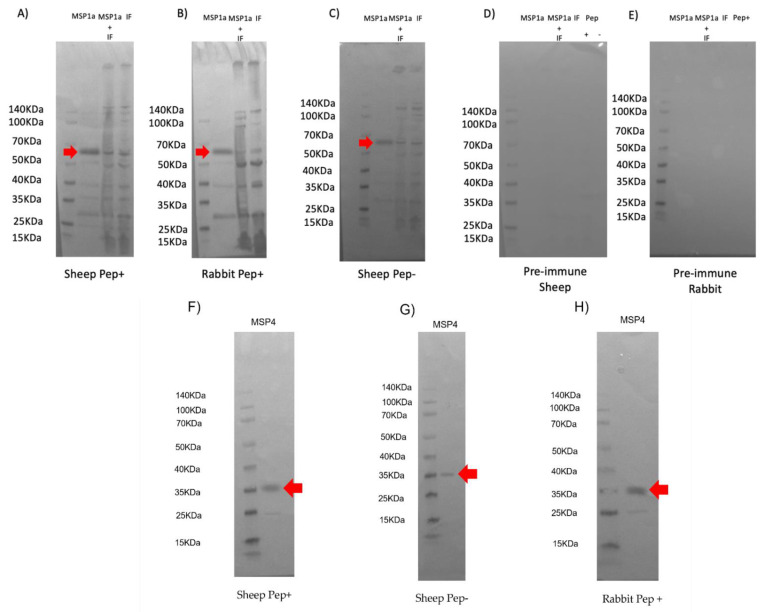
Antibody detection of the protein components included in the chimeric protein formulation by IgG antibodies produced in sheep and rabbit previously immunized with MSP1a and the chimeric protein exposed in the *E. coli* membrane. Samples equivalent to 10 µg of the total proteins: recombinant purified MSP1a (MSP1a), MSP1a expressed as an exposed antigen mixed with the insoluble *E. coli* fraction (MSP1a + IF), the insoluble *E. coli* fraction alone and recombinant purified MSP4 were loaded on a 12% polyacrylamide gel and for Western blot analysis, proteins were transferred to a nitrocellulose membrane as was explained above. (**A**) A Western blot was performed using IgG antibodies from sheep immunized with recombinant peptide+ (pep+) where MSP1a was detected showing a molecular weight of 67 kDa, as expected, and proteins from the insoluble fraction were also detected. (**B**) The same protein samples were also detected but using IgG antibodies from rabbit immunized with recombinant peptide+ and (**C**) sheep immunized with recombinant peptide-. (**D**) A Western blot using pre-immune serum samples from sheep and (**E**) rabbit were also used with the protein samples showing no specific signal. Red arrows denote the position of the antigen MSP1a. Spectra (Thermo Fisher Scientific) was used as molecular weight markers for protein electrophoresis, (**F**) recognition of recombinant MSP4 protein at the expected molecular weight of 35 KDa by IgG antibodies from sheep immunized with peptide+, (**G**) with peptide- and (**H**) IgG antibodies from rabbits immunized with peptide+.

To confirm the immunogenicity of all components of the chimeras formulation, additional Western blots were conducted to demonstrate the production of IgG antibodies against MSP1a, MSP4 and the insoluble fraction of *E. coli*. Antibodies against MSP1a were detected in both sheep and rabbits immunized with the recombinant chimeras, as well as against the insoluble *E. coli* proteins that were part of the formulation (Figure 3A–C and Figure S2). Specific signals were not detected when proteins were incubated with pre-immune serum (Figure 3D,E and Figure S2). Purified recombinant MSP4 [16] was detected by sera from Pep+ and Pep−immunized sheep as well as Pep+− immunised rabbits. This finding confirms that, again, the MSP4 peptides identified by the microarray method are present in the chimeras and remain immunogenic in the immunized animals (Figure 3F–H and Figure S2).

### 3.3. Inhibition of A. phagocytophilum Infection of Human HL60 and Tick ISE6 Cells 

**Figure 4:**
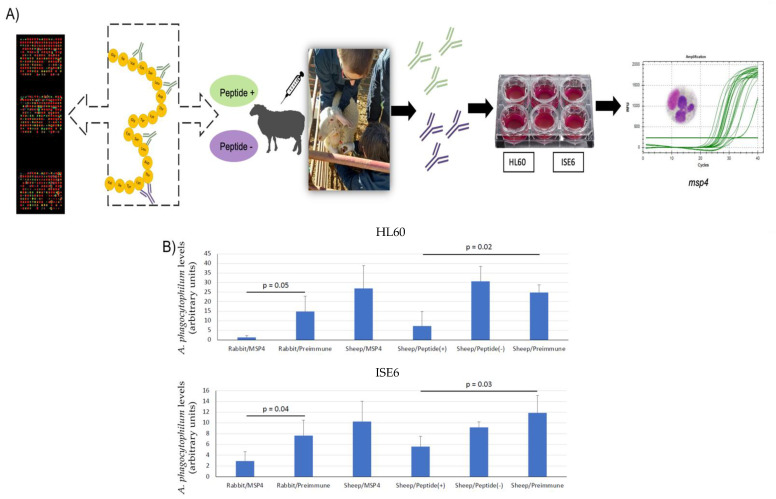
Experimental design in sheep. (**A**) mapping of protective epitopes with IgG from rabbits and lambs immunized with MSP4 [16]. Chimeric-antigens were designed based on protective epitopes identified by rabbit (Peptide+) and sheep (Peptide−) IgGs and produced for sheep immunization. The IgGs from the sera of immunized sheep were used to assess the efficacy of the protective response to *A. phagocytophilum* by inhibition assay in tick ISE6 and human HL60 cells; (**B**) normalized msp4 levels in treated HL60 and ISE6 cells in the antibody inhibition assay; role of antibodies against chimeric recombinant proteins from immunized sheep in the inhibition of *A. phagocytophilum* human NY18 infection of HL60 and ISE6 cells. Purified IgGs antibodies against *A. phagocytophilum* MSP4 from immunized rabbits and sheep were used to characterize the inhibition of pathogen infection of HL60 and ISE6 cells in comparison with the protective capacity of IgG from sheep immunized with the new MSP4 chimeric antigens (peptide+/peptide−) obtained from the MSP4 microarray analysis. Infection levels were determined by msp4 real-time PCR normalizing against human β-actin for HL60 cells and against tick rpS4 for ISE6 cells. Results were compared between groups treated with pre-immune/control and recombinant chimeric antigens antibodies by the Student’s *t*-test with unequal variance (*p* < 0.05; n = 3 replicates per treatment). Relative infection levels were expressed in arbitrary units.

An antibody inhibition assay using IgGs from sheep immunized with the chimeric antigens and from lambs and rabbits immunized with MSP4 from a previous study [16] was conducted to further characterize the antibody response and the potential protective capacity blocking the infection of *A. phagocytophilum* [33,34] of the new chimeric antigens containing predicted candidate protective (peptide+) and non-protective epitopes (peptide−) in immunized sheep in relation with the protective capacity of MSP4 (Figure 4A).

While rabbit IgG antibodies against *A. phagocytophilum* MSP4 recombinant protein inhibited pathogen infection of HL60 and ISE6 cells as in previous studies [16], IgGs from sheep immunized with peptide+ affected pathogen infection, but IgGs from sheep immunized with peptide− and IgGs from lambs immunized with MSP4 from a previous study did not protect against *A. phagocytophilum* infection (Figure 4B). These results suggest that the epitopes identified in the microarray assay using IgG antibodies from MSP4-immunized rabbits could be potentially protective in the sheep host.

**Figure 5:**
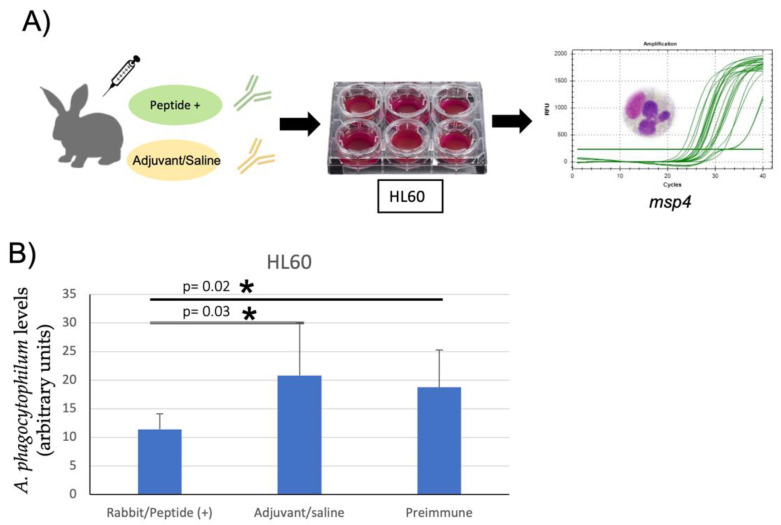
Experimental design of antibody inhibition assay in rabbits. (**A**) The IgGs from rabbits immunized with peptide+ and adjuvant/PBS alone were used to assess the efficacy of the protective response to *A. phagocytophilum* by inhibition assay in human HL60 cells in the conditions mentioned in the previous assay; (**B**) normalized msp4 levels in treated HL60 cells; antibody inhibition assay using IgGs from rabbits immunized with peptide + compared to rabbits immunized with adjuvant/saline and pre-immune sera. Results were analyzed using a Student’s *t*-test with unequal variance (* *p* < 0.05; n = 6 replicates per treatment). Asterisks denote statistically significant differences between groups. Relative infection levels were expressed in arbitrary units.

Furthermore, the inhibition assay in HL60 cells treated with IgG antibodies produced by peptide+-immunized rabbits showed a reduction in the infection of the cells with *A. phagocytophilum* when pathogen levels were compared by real-time PCR to cells treated with pre-immune antibodies and from saline-adjuvanted immunized rabbits. The IgG antibodies produced against the antigen peptide+ remained potentially protective in the rabbit host blocking the pathogen infection (Figure 5).

These results evidenced differences in the IgG response between MSP4 immunized rabbits and lambs and provided support that the design of new chimeric candidate protective antigens implementing quantum vaccinomics can improve the protective capacity of antigens in different hosts. In addition, future directions towards intelligent vaccine design should account for host-related factors, including age, sex, genetic factors, microbiota, pregnancy, and immune history which are factors that may influence vaccine efficacy, and also the optimization or personalization of vaccine dose, and use of adjuvants [35,36,37].

### 3.4. Anaplasma Species Peptide Sequence Alignment

Peptide sequence alignment showed the amino acid sequences from Anaplasma species with homology with the peptides identified in this study by microarray analysis (Table 2).

The protective peptides identified and selected for the design of chimeric antigens showed homology with amino acid sequences present in *Anaplasma* species. Additionally, in the putative conserved domains analysis, peptides 2 and 3 showed identity with sequence domains of a surface Ag 2 (pfam01617) that is a member of the porin superfamily (cl21487) and with a bacterial surface antigen present in *A. marginale*, *Ehrlichia chaffeensis*, *Rickettsia* spp. and *Wolbachia* spp. [38,39] (Figure S1). This result suggested a possible cross-protection against *A. marginale* and other tick-borne pathogens, but further experiments need to be conducted to confirm its efficacy.

Importantly, MSP4 has been characterized as a highly conserved protein in different *Anaplasma* strains with conserved epitopes and high antigenicity [38,40,41], and previous studies also showed serological cross-reactivity between *A. marginale* and *A. phagocytophylum* [42]. These findings suggest that this antigen may have a great potential for controlling infection of these pathogens that are the most important disease-producing pathogens of the genus *Anaplasma* [1,43]. 

One epitope (DGATGYAI) present in the peptide 2 sequence recognized by IgGs from MSP4 immunized rabbits was previously aligned to a region of *A. phagocytophylum* HSP70 (DGQTAVTI) and found as a B-cell reactive epitope [16]. HSP70 is a conserved protein implicated in infection processes [44,45] like MSP4, and is involved in tick–pathogen and host–pathogen interactions [14]. Furthermore, some serum cross-reactivity was found between these two proteins [16] probably caused by this common B-cell epitope present in peptide 2. The recognition of IgG antibodies against this epitope on MSP4 could be possibly identified by these antibodies on HSP70, present on the bacterial membrane, and may influence the control of *A. phagocytophylum* infection as evidenced in this study (Figure 4). However, further analysis of the epitopes recognized on *A. phagocytophylum* HSP70 by IgG antibodies against the MSP4 chimeric antigen, peptide+, would be necessary to confirm this hypothesis.

Quantum vaccinomics was proposed as a novel platform for the design and development of new chimeric antigens to achieve more effective vaccines by the combination of protective epitopes [17]. The characterization of molecular interactions between hosts and pathogens using omics technologies is also possible by identifying and characterizing immunogenic and protective epitopes as well as the domains in protein–protein interactions [46,47]. The application of the strategies proposed in this approach allowed the design and production of the chimeric vaccine antigen, peptide+, improving vaccine efficacy in the control of *A. phagocytophylum* infection in sheep, thus validating the application of this approach for the design of new and more effective vaccine candidates. 

Despite the relatively small percentage of linear B-cell epitopes, most methods have developed focusing on B-cell epitope predictions [48,49,50], and vaccine development research has received a growing interest in the ability to detect antibodies that recognized specific epitopes to predict vaccine efficacy [51,52]. However the main obstacle could be the 3D nature of the B-cell epitopes and that the current conformational B-cell epitope prediction methods are based on antigen structure-based prediction and mimotope-based prediction [53,54]. Comparing the prediction methods with quantum vaccinomics, this approach could be considered more reliable because, using antibodies from animals previously infected by a pathogen or exposed to a candidate antigen of interest, it is possible to identify epitopes or domains involved in vaccine protection. These identified domains could also provide information on cell interactome and regulome of vector–host–pathogen interactions. 

## 4. Conclusions

Although in previous studies MSP4 appeared not to be protective in sheep, contrary to evidence that suggested it might be an effective vaccine candidate for the control of anaplasmosis, the application of the tools provided by quantum vaccinomics resulted in the identification and combination of potential protective epitopes. The design of chimeric antigens allowed for more efficient epitope recognition by the immune system allowing peptides from MSP4 to be protective against *A. phagocytophylum* in different hosts. Nevertheless, additional in vivo experimental vaccination and infection trials in different hosts should be conducted to establish their efficacy.

## 5. Patents

The results of this study were part of the patent application by de la Fuente Garcia, J., Contreras Rojo, M. Vacuna para La prevención de la infección de *Anaplasma phagocytophilum* (Rickettsiales: Anaplasmataceae). Filing date: 30 September 2022. P202230845.

## Figures and Tables

**Table 1:** *A. phagocytophilum* MSP4 (GenBank ID: AFD54597) predicted immunogenic peptides reactive to IgGs from rabbit and sheep immunized with the recombinant protein.

IgGs host	Start	End	Peptide Sequence
Rabbit	14	48	AAVCACSLLISGSSFAYSGNNDASDVSGVMNGSFY
Rabbit	78	123	NKNLSTLNVSDPASFTQHDPSFKFAKSLLTSFDGATGYAIGGARV
Rabbit	165	180	KIDSVKDISVMLNAC
Rabbit	230	246	LIAGGSYHGIFDEQYA
Sheep	122	157	VEVEVGYKKFETLAESDYKHVESHNFVAVGRDATL

Peptides with Z-score > 2. Numbers indicate the position of the amino acid in the MSP4 protein sequence.

**Table 2:** Peptide sequence alignment using Blastp showing homologies with different Anaplasma species.

**Peptide 1:** AAVCACSLLISGSSFAYSGNNDASDVSGVMNGSFY	
**Organisms**	**Results**
*Anaplasma phagocytophilum* (taxid 948)	Hits: 135, Score: 70.5
Uncultured *Anaplasma* sp. (taxid 319051)	Hits: 21, Score: 70.1
*Anaplasma platys* (taxid 949)	Hits: 2, Score: 46.2
*Anaplasma ovis* (taxid 142058)	Hits: 1, Score: 40.4
*Anaplasma marginale* (taxid 770)	Hits: 146, Score: 37.4
**Peptide 2:** NKNLSTLNVSDPASFTQHDPSFKFAKSLLTSFDGATGYAIGGARV	
**Organisms**	**Results**
*Anaplasma phagocytophilum* (taxid 948)	Hits: 146, Score: 93.2
Uncultured *Anaplasma* sp. (taxid 319051)	Hits: 58, Score: 93.6
*Anaplasma marginale* (taxid 770)	Hits: 154, Score: 58.9
**Peptide 3:** KIDSVKDISVMLNAC	
**Organisms**	**Results**
*Anaplasma phagocytophilum* (taxid 948)	Hits: 137, Score: 51.5
Uncultured *Anaplasma* sp. (taxid 319051)	Hits: 56, Score: 51.5
*Anaplasma marginale* (taxid 770)	Hits: 146, Score: 31.6
**Peptide 4:** LIAGGSYHGIFDEQYA	
**Organisms**	**Results**
*Anaplasma phagocytophilum* (taxid 948)	Hits: 164, Score: 54.5
Uncultured *Anaplasma* sp. (taxid 319051)	Hits: 27, Score: 49.4
*Anaplasma* sp.	Hits: 4, Score: 39.2
*Anaplasma marginale* (taxid 770)	Hits: 144, Score: 36.7

Score: The highest alignment score calculated from the sum of the rewards for matched amino acids and penal ties for mismatches and gaps; Hits: Number of identified sequences.

## Supplementary Materials

The following supporting information can be downloaded at: https://www.mdpi.com/article/10.3390/vaccines10121995/s1, Figure S1: Surface Ag 2 alignment in A. marginale, Ehrlichia chaffeensis, Rickettsia spp., Wolbachia spp with Peptide 2 and Peptide 3. Figure S2: Entire Western blots including the intensity ratio of each band that was relevant in the study.
