# Supplementary material for: A Quantum Vaccinomics Approach for the Design and Production of MSP4 Chimeric Antigen for the Control of Anaplasma phagocytophilum Infections
Source: Vaccines (Basel). 2022 Nov 24;10(12):1995. doi: 10.3390/vaccines10121995 (PMC9784196; doi:10.3390/vaccines10121995)
Supplement: Supplementary file 1 [file vaccines-10-01995-s001.zip › vaccines-1997461-supplementary.pptx]

## Slide 1
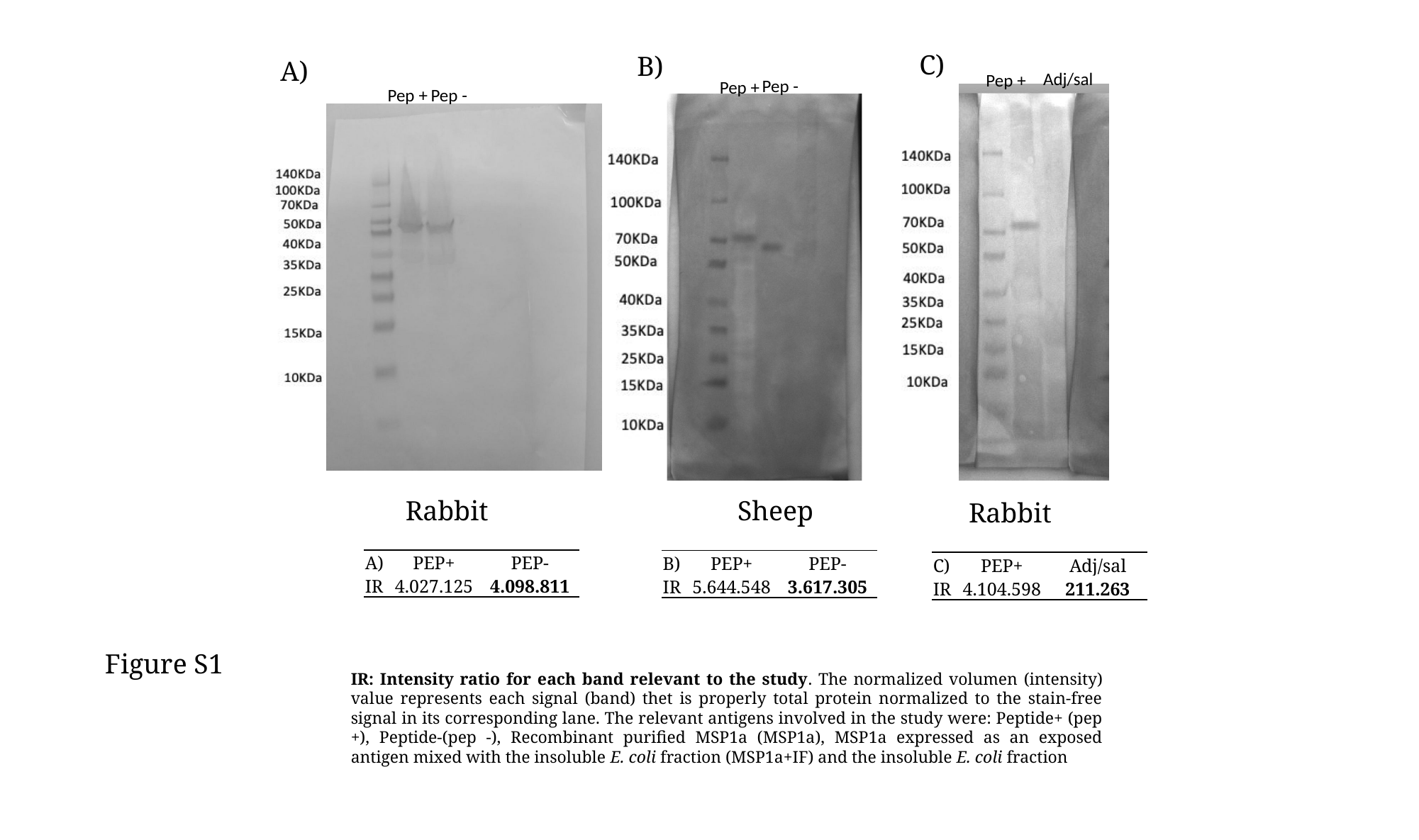

C)
B)
A)
Adj/sal
Pep +
Pep -
Pep +
Pep +
Pep -
Sheep
Rabbit
Rabbit
| A) | PEP+ | PEP- |
| --- | --- | --- |
| IR | 4.027.125 | 4.098.811 |
| B) | PEP+ | PEP- |
| --- | --- | --- |
| IR | 5.644.548 | 3.617.305 |
| C) | PEP+ | Adj/sal |
| --- | --- | --- |
| IR | 4.104.598 | 211.263 |
Figure S1
IR: Intensity ratio for each band relevant to the study. The normalized volumen (intensity) value represents each signal (band) thet is properly total protein normalized to the stain-free signal in its corresponding lane. The relevant antigens involved in the study were: Peptide+ (pep +), Peptide-(pep -), Recombinant purified MSP1a (MSP1a), MSP1a expressed as an exposed antigen mixed with the insoluble E. coli fraction (MSP1a+IF) and the insoluble E. coli fraction

## Slide 2
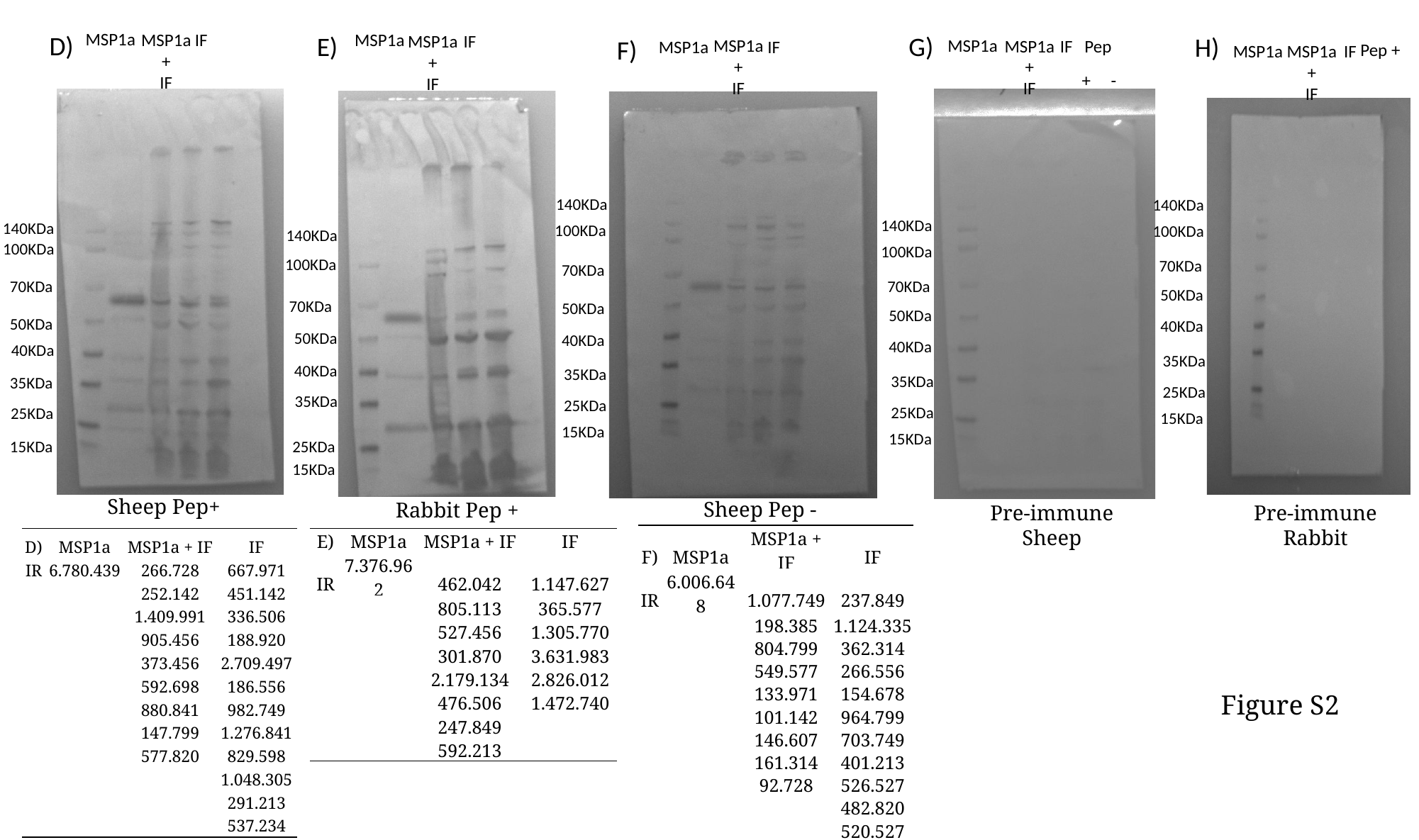

MSP1a
MSP1a
+
IF
IF
MSP1a
D)
E)
G)
H)
MSP1a
+
IF
IF
F)
MSP1a
+
IF
MSP1a
MSP1a
+
IF
IF
Pep
MSP1a
IF
Pep +
MSP1a
MSP1a
+
IF
 IF
+ -
140KDa
140KDa
140KDa
140KDa
100KDa
100KDa
140KDa
100KDa
100KDa
100KDa
70KDa
70KDa
70KDa
70KDa
50KDa
70KDa
50KDa
50KDa
50KDa
40KDa
50KDa
40KDa
40KDa
40KDa
35KDa
40KDa
35KDa
35KDa
35KDa
25KDa
35KDa
25KDa
25KDa
25KDa
15KDa
15KDa
15KDa
15KDa
25KDa
15KDa
Sheep Pep+
Sheep Pep -
Rabbit Pep +
Pre-immune Sheep
Pre-immune Rabbit
| F) | MSP1a | MSP1a + IF | IF |
| --- | --- | --- | --- |
| IR | 6.006.648 | 1.077.749 | 237.849 |
| | | 198.385 | 1.124.335 |
| | | 804.799 | 362.314 |
| | | 549.577 | 266.556 |
| | | 133.971 | 154.678 |
| | | 101.142 | 964.799 |
| | | 146.607 | 703.749 |
| | | 161.314 | 401.213 |
| | | 92.728 | 526.527 |
| | | | 482.820 |
| | | | 520.527 |
| | | | 209.263 |
| E) | MSP1a | MSP1a + IF | IF |
| --- | --- | --- | --- |
| IR | 7.376.962 | 462.042 | 1.147.627 |
| | | 805.113 | 365.577 |
| | | 527.456 | 1.305.770 |
| | | 301.870 | 3.631.983 |
| | | 2.179.134 | 2.826.012 |
| | | 476.506 | 1.472.740 |
| | | 247.849 | |
| | | 592.213 | |
| D) | MSP1a | MSP1a + IF | IF |
| --- | --- | --- | --- |
| IR | 6.780.439 | 266.728 | 667.971 |
| | | 252.142 | 451.142 |
| | | 1.409.991 | 336.506 |
| | | 905.456 | 188.920 |
| | | 373.456 | 2.709.497 |
| | | 592.698 | 186.556 |
| | | 880.841 | 982.749 |
| | | 147.799 | 1.276.841 |
| | | 577.820 | 829.598 |
| | | | 1.048.305 |
| | | | 291.213 |
| | | | 537.234 |
Figure S2

## Slide 3
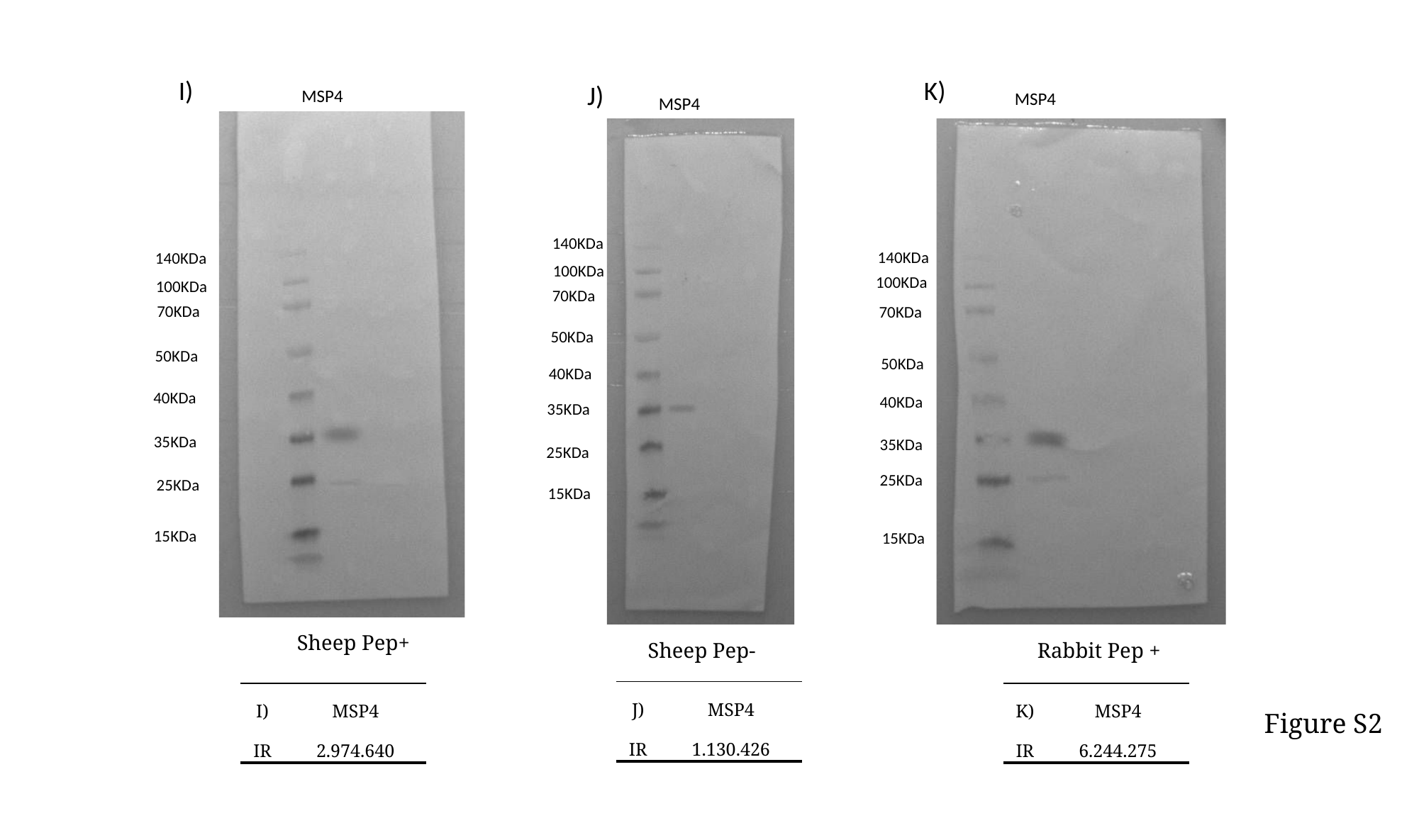

I)
K)
J)
MSP4
MSP4
MSP4
140KDa
140KDa
140KDa
100KDa
100KDa
100KDa
70KDa
70KDa
70KDa
50KDa
50KDa
50KDa
40KDa
40KDa
40KDa
35KDa
35KDa
35KDa
25KDa
25KDa
25KDa
15KDa
15KDa
15KDa
Sheep Pep+
Sheep Pep-
Rabbit Pep +
| J) | MSP4 |
| --- | --- |
| IR | 1.130.426 |
| I) | MSP4 |
| --- | --- |
| IR | 2.974.640 |
| K) | MSP4 |
| --- | --- |
| IR | 6.244.275 |
Figure S2
